# Ectopic ureter opening into the vagina in an adult with duplex kidney: a case report and literature review

**DOI:** 10.3389/fsurg.2026.1871517

**Published:** 2026-06-23

**Authors:** Haigang Luo, Wenting Zhang, Xianjun Sun, Wei Bian

**Affiliations:** 1 Department of Radiology, Central Health Center of Daqiao Town, Jiaxing, Nanhu District, China; 2 Department of Radiology, Jiaxing Maternity and Child Health Care Hospital, College of Medicine, Jiaxing University, Jiaxing, China; 3 Department of Urology, Jiaxing Maternity and Child Health Care Hospital, College of Medicine, Jiaxing University, Jiaxing, China

**Keywords:** duplex kidney, ectopic ureter, magnetic resonance urography, renal colic, vaginal discharge

## Abstract

**Introduction:**

Duplex kidney with an ectopic ureter typically presents with continuous urinary incontinence in childhood. Adult females presenting with renal colic and intermittent vaginal discharge are rare, especially when initial ureteroscopy is normal.

**Methods:**

We report a 26-year-old woman with a one-week history of severe right flank pain, each episode accompanied by a small amount of clear, odourless vaginal discharge. She had undergone caesarean section four months earlier. Non-contrast computed tomography (CT), magnetic resonance imaging (MRI) and magnetic resonance urography (MRU) were performed, followed by ureteroscopy and laparoscopic exploration.

**Results:**

Non-contrast CT suggested a right duplex kidney with dilatation of the upper-pole ureter but could not identify the distal termination. MRU clearly demonstrated a complete right duplex system with a markedly dilated and tortuous upper-pole ureter that did not connect to the bladder; T2-weighted fat-saturated images showed the terminal ureter entering the vaginal region. Ureteroscopy revealed a normal-appearing ipsilateral ureteric orifice. Laparoscopy identified a 2-cm diameter ectopic ureter behind the broad ligament, with its distal opening into the vagina. Laparoscopic ligation of the ectopic ureter followed by ureteroneocystostomy resulted in complete resolution of symptoms.

**Discussion:**

In adult women presenting with renal colic and intermittent vaginal discharge, a duplex kidney with an ectopic ureter should be suspected even when ureteroscopy is normal. Non-contrast MRI combined with MRU is the best non-invasive imaging method for diagnosis.

## Introduction

Duplex kidney is a common congenital anomaly of the urinary tract, with a reported incidence of approximately 0.8%. An ectopic ureter is most frequently associated with the upper pole moiety and, in females, may open into the urethra, the vestibule, or the vagina (1). The classic clinical presentation is continuous urinary leakage unrelated to posture or voiding (2). However, symptomatic presentation in adulthood, particularly with episodic renal colic and intermittent vaginal discharge, is very unusual.

When the ectopic orifice is narrow and opens into the vagina, the patient may not experience classical continuous leakage; instead, vaginal discharge occurs only during episodes of renal colic. This atypical pattern is easily mistaken for a gynaecological disorder, a postoperative complication, or simple renal colic. Moreover, when routine cystoscopy or ureteroscopy shows normal ureteric orifices, the diagnosis of a duplex system is often dismissed, leading to prolonged delay.

We report a case of right duplex kidney with an ectopic ureter opening into the vagina in a 26-year-old woman who presented with renal colic and pain-related vaginal discharge, with false-negative ureteroscopy.

## Case presentation

A 26-year-old woman presented with a one-week history of sudden, severe right flank pain. The pain was colicky in nature, radiated to the right lower abdomen, and worsened in paroxysms. During each severe episode, she noticed a small amount of clear, odourless vaginal discharge. There was no urgency, frequency, dysuria, gross haematuria, continuous leakage, fever, or chills. Treatment with antibiotics and antispasmodics at another hospital had been unsuccessful.

She had undergone appendicectomy 10 years earlier and a caesarean section four months before presentation. She had one child (G1P1). Her menstrual cycles were regular, with the last menstrual period on 7 March 2026.

Physical examination revealed a temperature of 37.5 °C; other vital signs were normal. The abdomen was soft and non-tender. There was right renal angle tenderness and marked right costovertebral angle tenderness. Gynaecological examination showed a normal vulva and vagina, with a small amount of clear fluid in the vaginal vault. The cervix was smooth, and the uterus and adnexae were unremarkable.

## Imaging findings

**Figure 1:**
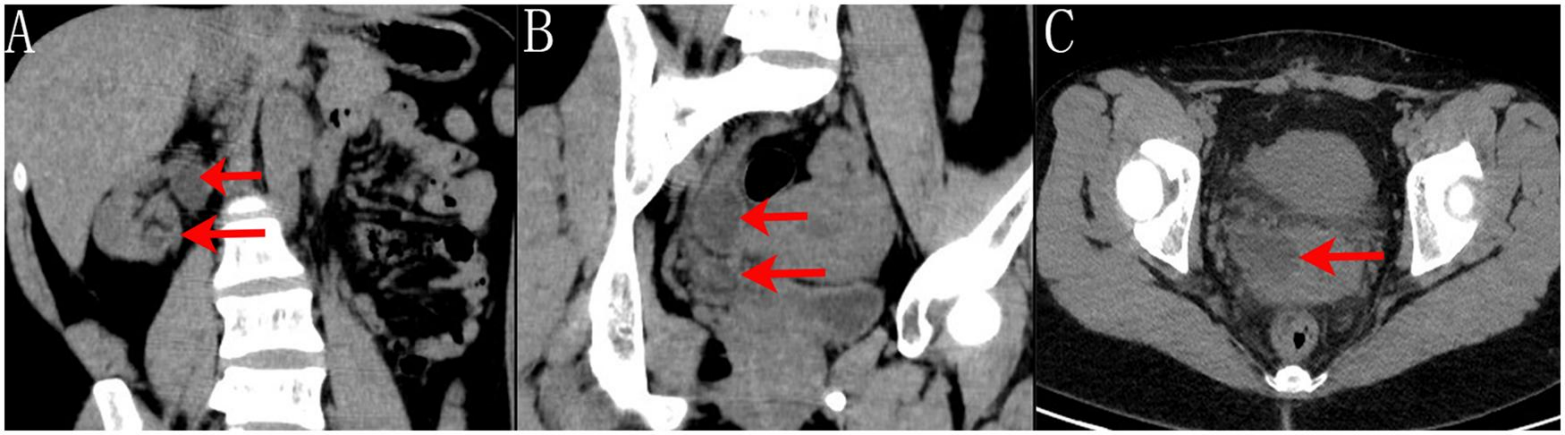
Unenhanced CT of the urinary tract. **(A,B)** Oblique coronal reformatted images demonstrate a duplex kidney with ureteral duplication. The upper pole moiety and corresponding ureter are dilated (short arrows), while the lower pole moiety and ureter are normal (long arrows). **(C)** Axial image shows the dilated distal ureter adjacent to the vagina.

Non-contrast CT demonstrated a right duplex kidney with dilatation of the upper-pole ureter, which was tortuous in its lower part (Figures 1A–C). The appearance suggested a duplex system, but the exact distal termination of the dilated ureter could not be identified.

**Figure 2:**
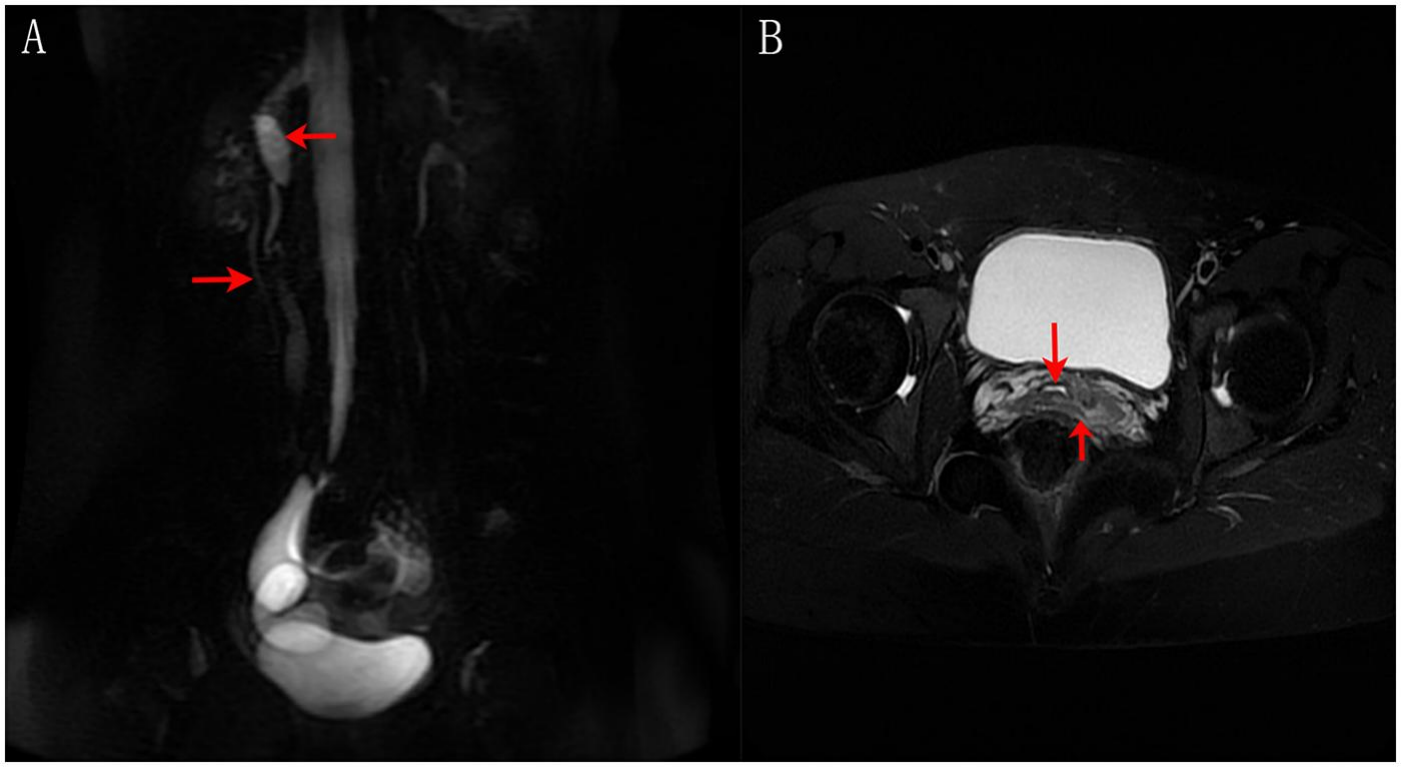
MRI of the urinary tract. **(A)** MR urography (MRU) shows a duplex kidney with ureteral duplication. The upper pole moiety and ureter are dilated (short arrows), and the lower pole moiety and ureter are normal (long arrows). **(B)** Axial T2-weighted fat-suppressed (T2WI-FS) image reveals tapering of the distal dilated ureter (long arrow) and its ectopic opening into the vagina (short arrow).

Magnetic resonance imaging (MRI) and MR urography (MRU) were subsequently performed. MRI was performed using a 1.5 T scanner (GE SIGNA Voyager) without intravenous contrast. The protocol included: axial T2-weighted fat-saturated with PROPELLER (PROP) sequence (TR/TE not specified, slice thickness 4 mm), coronal T2-weighted fat-saturated (slice thickness 3 mm), and 3D MR urography using a 3D fast recovery fast spin-echo (FRSE-XL) sequence with fat saturation. The 3D MRU parameters were: slice thickness 2.0 mm, field of view 38 cm, matrix resulting in pixel size 1.2 × 1.7 mm, effective TE approximately 560–1,000 ms, echo spacing 5.9 ms, and respiratory triggering. Maximum intensity projection (MIP) reconstructions were generated from the 3D dataset. Coronal T2-weighted images clearly demonstrated two separate renal pelvises on the right – the “double pelvis sign”. The upper pelvis connected to a widely dilated and tortuous ureter measuring 1.5–2 cm in diameter. Three-dimensional MRU reformats showed two complete ureters on the right: the lower-pole ureter was of normal calibre and drained normally into the bladder trigone; the upper-pole ureter was dilated throughout, ended in a saccular appearance on MRU, and did not communicate with the bladder. T2-weighted fat-saturated images showed the dilated ureter passing towards the retro-uterine space and the upper vagina, with a fine distal opening into the vagina (Figures 2A,B). A diagnosis of right duplex kidney with an upper-pole ectopic ureter opening into the vagina was made on the basis of the MRI/MRU findings.

## Ureteroscopy

Ureteroscopy revealed a normal-appearing ureteric orifice on each side, with clear efflux. A guidewire was advanced into the right ureter up to the renal pelvis, with no abnormality detected.

## Laparoscopic exploration and treatment

Laparoscopy identified a large, 2-cm-diameter dilated ureter behind the broad ligament on the right, separate from the normal ipsilateral ureter. The distal end of this ectopic ureter opened directly into the vagina.

The patient underwent laparoscopic ligation of the distal ectopic ureter followed by ureteroneocystostomy, with placement of two ureteric stents. Post-operatively, renal colic resolved completely and vaginal discharge stopped. CT on day 6 showed relief of the right-sided obstruction, and she was discharged on day 7. At the time of manuscript submission, the patient had been followed for 1 month post-operatively, with no recurrence of symptoms and normal serum creatinine. Longer-term follow-up is ongoing.

Written informed consent was obtained from the patient for publication of this case report and any accompanying images.

## Discussion

This case has several notable features that merit discussion. This is a narrative (non-systematic) literature review. To identify relevant cases, we searched PubMed and Google Scholar up to April 2026 using the terms “ectopic ureter”, “vaginal ectopic ureter”, and “adult”. Reference lists of retrieved articles were also screened. We included case reports of female patients aged ≥18 years with a vaginal ectopic ureter who did not present with classic continuous incontinence as the predominant symptom. Typical childhood-onset cases were excluded. A PRISMA-compliant flow diagram is not provided given the narrative scope of this review. To better understand the spectrum of adult-onset vaginal ectopic ureters and to position our case within the existing literature, we reviewed previously reported cases in adult women (>18 years) who did not present with classic continuous incontinence. Table 1 summarizes these cases, which are discussed in detail below in relation to each key clinical aspect of our case.

**Table 1:** Adult cases of vaginal ectopic ureter without classic continuous incontinence reported in the literature.

Author, Year	Age	Presentation	Diagnostic Modality	Treatment	Outcome
Current case, 2026	26	Renal colic + intermittent vaginal discharge	MRU	Ureteroneocystostomy	Symptom resolution
Hantman, (1983) (3)	28	Fever, flank pain (pyelonephritis). No incontinence.	Ultrasound, Antegrade pyelography	Heminephrectomy, ureterectomy	Full recovery
Andersen et al. (1986) (8)	24	Extra-uterine gestation, pelvic pain. No incontinence.	Fistulography, HSG	Nephroureterectomy, hysterectomy	Not specified^a^
Cisternino et al. (1988) (5)	40	Lateralized lumbar pain during intercourse. Incontinence after second pregnancy.	IVP, CT, DMSA	Nephroureterectomy	Incontinence resolved
Engelstein et al. (1996) (4)	42	Fever, chills, flank pain. No incontinence.	Anterograde nephrostogram, Retrograde pyelography	Upper pole nephroureterectomy	Uneventful recovery
Sheih et al. (1997) (9)	22	Intermenstrual vaginal discharge (since menarche), foul purulent discharge.	Ultrasound, MRI	Vaginal septum excision (renal surgery planned)	Awaiting renal surgery
Leonovicz et al. (1997) (10)	24	Recurrent UTIs, persistent purulent vaginal discharge.	CT, Excretory urography	Distal ureterectomy	Asymptomatic (cyst remains)^c^
Sameshima et al. (2005) (7) (Case 1)	65	Vaginal adenocarcinoma (incidental finding). Asymptomatic for ureter.	Imaging, Histology	Radical hysterectomy, nephroureterectomy	Not specified for incontinence^a^
Sameshima et al. (2005) (7) (Case 2)	38	Cervical squamous cell carcinoma (incidental). No incontinence.	Pre-op imaging	Radical hysterectomy	Post-op vesicovaginal fistula^b^
Kumar et al. (2007) (6)	22	Menouria (menstrual blood in urine), infertility. No vaginal menstruation.	MRI, Cystoscopy	Nephroureterectomy, fistula repair, septum excision	Normal menstruation, continent
Dueñas-Garcia and Hall, (2016) (11)	38	Copious vaginal fluid at 31 weeks gestation (simulated PPROM). Symptoms began in pregnancy.	CT urogram, MRI urogram	Robotic uretero-ureteral anastomosis	Symptom resolution

UTI, urinary tract infection; IVP, intravenous pyelography; HSG, hysterosalpingography; CT, computed tomography; MRI, magnetic resonance imaging; MRU, magnetic resonance urography; PPROM, preterm premature rupture of membranes.

a Outcome not reported in the source publication.

b A vesicovaginal fistula occurred postoperatively; the patient's continence status was not reported.

c No subsequent publication reporting completion of renal surgery was identified.

### Clinical presentation

This presentation pattern differs from previously reported adult cases of vaginal ectopic ureter (Table 1). For instance, some adults presented with recurrent pyelonephritis (3, 4), post-coital pain (5), menouria (6), or were asymptomatic and diagnosed incidentally during malignancy work-up (7). Notably, only one other case described symptom onset after pregnancy (5), and none reported pain-related intermittent discharge as the dominant feature. Regarding the timing of symptom onset, our patient remained asymptomatic until after caesarean section. Whether this represents a causal relationship or mere temporal coincidence is unknown. It is speculative that pregnancy-related anatomical changes or post-operative adhesions might have altered ureteral dynamics or unmasked a previously subclinical condition. No direct evidence supports this hypothesis. Thus, our case expands the clinical spectrum of this condition in adults. We hypothesize that the intermittent, pain-related vaginal discharge resulted from acute distention of the obstructed upper-pole ureter during renal colic, which increased intraluminal pressure and forced transient leakage through a normally stenotic ectopic orifice. This proposed mechanism, while physiologically plausible, remains speculative and has not been experimentally validated.

### Diagnostic pitfalls

The diagnostic challenge is not unique to our patient. As shown in Table 1, several adult cases required multiple imaging modalities (ultrasound, CT, IVP, cystoscopy) before the diagnosis was established, and in some patients the ectopic orifice was only identified intraoperatively (9, 10). This further supports our conclusion that a high index of suspicion is required even when routine investigations are normal. Non-contrast CT in this case provided initial clues but is limited in its ability to display the distal course of the ectopic ureter. Published data indicate a sensitivity of about 78% for detecting duplication on CT in humans (12). However, direct visualization of the ectopic orifice itself remains poor with non-contrast CT, often necessitating cross-sectional imaging such as MRU (13).

### Imaging recommendations

This case illustrates the key advantages of MRU. As a water-based imaging technique, MRU is non-invasive, free of ionising radiation, and does not require iodinated contrast material. Dilated ureters are naturally visualised, and three-dimensional reconstructions display the entire ureteric course and its termination. MRU clearly distinguishes the upper (ectopic) from the lower (orthotopic) ureter. The superiority of MRU in this setting is further illustrated by several previously reported adult cases (Table 1), where MRU or MR urography successfully delineated the ectopic ureter and its vaginal insertion (6, 9, 11). In contrast, cases diagnosed before the widespread availability of MRU often required more invasive procedures such as antegrade pyelography or surgical exploration (3, 5). In this patient, unenhanced MRI plus MRU directly demonstrated three diagnostic signs: two ureters, an ectopic ureter that did not connect to the bladder, and its termination in the vaginal region. Recent studies confirm that MRU has significantly better accuracy than conventional imaging in complex urinary tract anomalies (13, 14). If preoperative contrast-enhanced CT with a delayed phase had been performed, it would likely have demonstrated contrast agent passing into the vagina, further aiding the diagnosis of this condition.

### Embryological basis: the Weigert-Meyer rule

The anatomical findings in this case conform to the Weigert-Meyer rule, which describes the typical relationship in complete ureteral duplication (15). This rule states that the ureter from the upper pole moiety inserts inferomedially to the lower pole ureter and is often ectopic, while the lower pole ureter inserts orthotopically but is prone to reflux. In our patient, the dilated upper-pole ureter opened ectopically into the vagina, while the lower-pole ureter drained normally into the bladder trigone, perfectly illustrating this embryological principle.

### Treatment considerations

Because the upper-pole moiety in this patient was judged to have preserved function based on intraoperative appearance (grossly normal parenchyma with good thickness) and preoperative MRU findings (well-preserved renal parenchyma without significant thinning or scarring), and because she was a young woman, ureteroneocystostomy with distal ligation was chosen to preserve functioning renal tissue. We acknowledge that no quantitative functional assessment (e.g., DMSA scan) was performed, which is a limitation of this report (see Limitations).

Our choice of ureteroneocystostomy contrasts with the ablative procedures performed in most previously reported adult cases (Table 1). Arguments in favor of reconstruction include preservation of maximal functional nephron mass in a young patient, avoidance of the long-term risks of reduced renal reserve, and prevention of the need for a more extensive renal resection. Arguments against reconstruction include the potential for persistent reflux or obstruction at the new anastomosis, and the possibility of leaving behind dysplastic tissue that could cause future complications. In our patient, the complete symptom resolution and post-operative imaging confirmation of obstruction relief validate our approach, which is also supported by a recent systematic review favoring reconstructive surgery over resection when the upper moiety retains useful function (16).

## Limitations

This study has several limitations. First, as a single case report, the findings are not generalizable. Second, no quantitative functional imaging (e.g., DMSA scan) was performed to objectively confirm preserved function of the upper-pole moiety. Third, long-term follow-up data beyond the immediate post-operative period are unavailable. Fourth, the proposed pathophysiological mechanisms (e.g., “pressure-driven intermittent leakage”, post-caesarean symptom onset) remain speculative and require further validation.

In conclusion, although rare, a duplex kidney with a vaginally inserted ectopic ureter can present in adulthood without classic continuous incontinence, instead manifesting as renal colic with pain-related vaginal discharge. Non-contrast CT may provide suspicious findings but is not diagnostic, and ureteroscopy can be falsely negative. Non-contrast MRI combined with MRU is the best non-invasive imaging technique for this condition and should be the first-line examination. CT urography (CTU) is also capable of depicting the entire ureteric course, and delayed-phase contrast-enhanced CT can demonstrate contrast medium passing into the vagina, which aids in confirming the diagnosis. Laparoscopic ureteroneocystostomy is a safe and effective treatment that preserves renal function.

## Data Availability

The original contributions presented in the study are included in the article/Supplementary Material, further inquiries can be directed to the corresponding author.
